# The Role of E-Content Development in Medical Teaching: How Far Have We Come?

**DOI:** 10.7759/cureus.43208

**Published:** 2023-08-09

**Authors:** Maithili N Bankar, Nandkishor J Bankar, Brij Raj Singh, Gulshan R Bandre, Yogendra P Shelke

**Affiliations:** 1 Anatomy, Datta Meghe Medical College, Datta Meghe Institute of Higher Education and Research, Wardha, IND; 2 Microbiology, Jawarhal Nehru Medical College, Datta Meghe Institute of Higher Education and Research, Wardha, IND; 3 Microbiology, Bhaktshreshtha Kamalakarpant Laxman Walawalkar Rural Medical College, Ratnagiri, IND

**Keywords:** technology, deep learning, medical teaching, interactive elements, simulations, multimedia, digital content, e-learning, e-content development

## Abstract

With the advancements in technology, medical educators are now able to create and deliver content to students through digital platforms. Electronic content (e-content) development has allowed educators to incorporate multimedia, animations, simulations, and interactive elements which support verbal instruction, such as improved expression and comprehension, into their teaching materials. E-content development is a relatively new field, but it is growing very rapidly. Recent findings have indicated that the e-learning sector will likely experience a huge surge in the upcoming years. The Indian government has launched various initiatives for e-content development in medical education. E-content development has great potential and can be used in various learning scenarios. While it initially gained popularity in higher education, it has since been applied to many other sectors, including healthcare. It allows educators to create highly engaging learning experiences that are accessible by all students. Challenges in e-content development include availability of the internet, creating content that is engaging and relevant to a wide range of learners, and access. Still, it is expected that the use of e-content in medical teaching will continue to increase in the future. The future of e-content development in medical teaching is likely to see continued growth and innovation as technology advances and more educators and learners recognize the benefits of online and digital resources.

## Introduction and background

E-content development is creating digital content for online platforms, such as videos, animations, simulations, interactive quizzes, and interactive activities [1]. E-content is explicitly created for digital platforms and should not be repurposed for paper-based use [2]. The content can be delivered to students through online platforms such as digital interactive whiteboards (e-iWBs) and digital learning environments (DLEs) [3]. E-content development is a relatively new field, but it is proliferating, and findings have indicated that this sector is likely to experience a massive surge in the upcoming years [4,5]. E-Content Development has much potential and can be used in various learning scenarios [6], which are best used in flipped classrooms, blended learning, and virtual classrooms and can also be used to supplement existing instructional materials or create new ones [7].

The past ten years have seen significant progress in using e-learning materials for medical education [8,9]. The world has changed drastically due to the coronavirus disease (COVID-19) pandemic. New rules, technologies, and institutions are leading to further transformations. In contrast, digital technologies such as Artificial Intelligence (AI), deep learning (DL), and machine learning (ML) are being used in the medical field, and specialized educational content is being released freely on platforms. With technological advancements, medical educators can create and deliver content to students through digital platforms [10,11]. This has enabled educators to provide students with more engaging, interactive, and insightful learning experiences. Electronic content (e-content) development has allowed educators to incorporate multimedia, animations, simulations, and interactive elements that support verbal instruction, such as improved expression and comprehension, into their teaching materials [12,13]. This has improved the learning experience and made it easier for students to comprehend complex topics. As a result, e-content development has become an integral part of medical teaching today [14-16]. E-content development has enabled medical educators to incorporate online quizzes and assessments into their teaching materials. This helps to assess students’ understanding of the material and provides feedback on their performance [17]. Furthermore, e-content development has enabled medical educators to create customized content for specific courses or subjects [18]. The beauty of e-content is that it breaks down barriers of time and place, enabling individuals to pursue learning independently [17,18]. For example, some courses, such as anatomy or physiology, may require more visual elements than others. E-content development allows for these visual elements to be incorporated into the teaching materials in an engaging and informative way [19]. Also, it enables medical educators to tailor their content according to the needs of their students [20,21].

Medical education is an essential part of ensuring the quality of healthcare [17]. It is the dynamic structural element of good education, and therefore, this article aims to explore how far we have come regarding e-content development and its role in medical teaching.

## Review

Methods

To conduct a comprehensive literature search, we used the following databases: PubMed, MEDLINE, Scopus, and Google Scholar. We searched for articles published between 2013, and 2023, using the following search terms: ("e-content" OR "e-learning" OR "medical education" OR "medical teaching" OR "online learning" OR "digital education") AND ("curriculum" OR "content development" OR "instructional design" OR "courseware" OR "technology-enhanced learning"). We applied the following inclusion criteria for the final review: (1) original research articles, (2) English language, (3) peer-reviewed, (4) relevant to e-content in medical education, (5) full-text available, and (6) published in the specified time frame.

Articles Screened

After conducting the initial search, we identified a total of 2,558 articles across the searched databases. We then excluded duplicates (n=451) and conducted an initial screening of titles and abstracts, which excluded a further 1,943 articles. After the full-text screening of the remaining 164 articles, we excluded 146 articles for not meeting the inclusion criteria either they were not related to e-content or some were for patient care, leaving a total of 18 articles for the final review [22].

Duration and Number of Articles Included in Final Review

**Figure 1:**
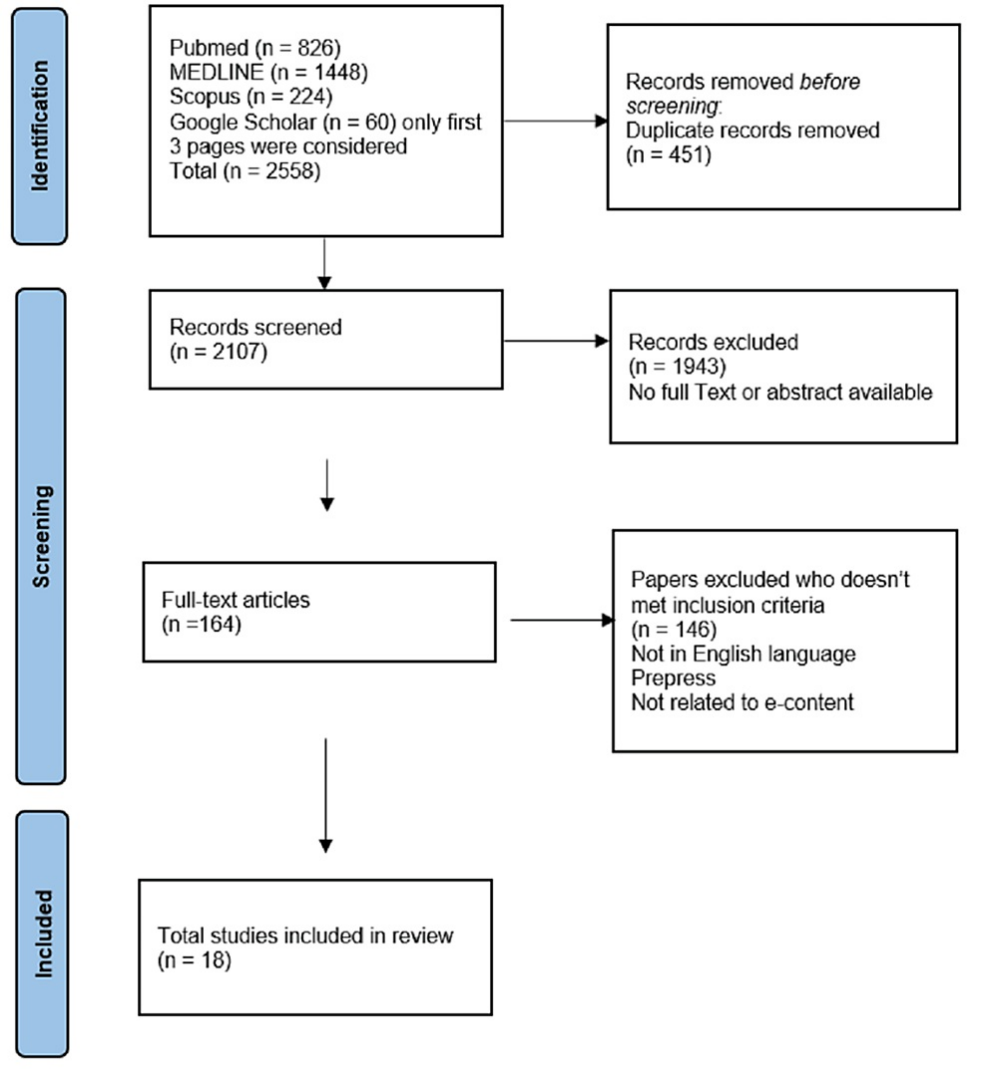
Prisma flow chart n - Number of Studies

The literature search was conducted in February 2023. The final review included a total of 18 articles from the years 2013 to 2023 (Figure 1).

Articles included in the review were, each of e-books, mixed reality, mobile e-content, online multiple-choice questions (MCQ), quizzes, and simulations. Two articles each were based on virtual teaching and web-based teaching. Three articles were on the e-learning platform and five were based on video-based e-learning (Table 1). 

**Table 1:** Articles included in the study MCQ - Multiple Choice Question, HTML - Hypertext Markup Language

Sr No	Authors	Year	E-content used or developed	Methods	Method of assessment	Findings
1	Tang F et al. [23]	2017	Video	One group watched a recorded lecture and video before the lecture and the other group was assigned traditional lecture-based teaching.	Pre- and post-tests	Promising results in ophthalmology by flipped classroom teaching
2	Ji M et al. [24]	2022	Video	Recorded video links to one group and traditional teaching to another group	Physiology final exam papers	Flipped classroom shows promising results in a physiology course
3	Baratz G et al. [25]	2022	Mixed reality	One group completed a mixed reality module and the cadaveric dissection and the other group only dissection	Quiz and survey and final examination	mixed reality improves long-term retention in breast anatomy
4	Glosser LD et al. [26]	2022	Video	Video before simulation and video in between simulation in two gropus	Assessed by a 44-point standardized checklist	The use of video instruction has been found to significantly improve students' clinical performance compared to relying solely on simulation-based learning.
5	Co M et al. [27]	2021	Video	Web-based surgical skill learning sessions and conventional face-to-face teaching in two groups	Objective Structured Assessment of Technical Skills Global rating scale	Surgical skills were comparable with online web-based teaching and face-to-face teaching
6	Lee LA et al. [28]	2018	Mobile e-content	Interactive multimedia versus Microsoft PowerPoint show	MCQ and multimedia situation tests	Mobile e-learning offers a flexible and interactive learning experience
7	Chang TP et al. [29]	2014	E-learning	Web-based, interactive, peer-reviewed Flash/HTML5 modules and traditional teaching	Post-rotation testing and in-training examination	Asynchronous E-learning is a promising modality
8	Gruner D et al. [30]	2015	E-learning	E-learning and articles (peer-reviewed)	Pre- and post-knowledge quiz and self-assessment	Improved global health knowledge scores
9	Davids MR et al. [31]	2014	E-learning	Comparison of two iterations of e-learning	Subjective, self-reported, and objective data	The design-test-redesign approach had significant improvements
10	Taveira-Gomes T et al. [32]	2015	Quiz	Study quiz and quiz groups	Recall accuracy is graded using a 4-point Likert scale	The study quiz task had a high impact on recall accuracy.
11	Dean WH et al. [33]	2021	Simulation	Simulation-based cataract surgical training and conventional training	Validated competency assessment rubric	Rapid acquisition of surgical competence
12	Yang C et al. [34]	2020	Web based	Flipped classroom web-based learning system for anatomy and traditional teaching	5-point Likert scale questionnaire	The flipped classroom is an effective learning tool
13	Moazami F et al. [35]	2014	Virtual	Virtual and traditional learning	Multiple Choice Questions (MCQ) and Essay Questions	More effective as compared to lecture-based training
14	Hsiao CC et al. [36]	2016	E-books	Interactive Multimedia eBooks and traditional PowerPoint	Pre-test and post-test	Interactive multimedia eBooks are more effective
15	Seifert LB et al. [37]	2020	Video	standardized teaching videos and traditional method	Structured checklists	Preferred method
16	Boscolo-Berto R et al. [38]	2021	Virtual	Virtual and traditional learning	Post-test	Significant improvement in the combination of virtual to traditional gross dissection
17	Mitra NK et al. [39]	2015	Online MCQ	Online and paper-based MCQ	Summative MCQ test scores	Computer-based formative tests with automated feedback led to improved performance.
18	Carpenter R et al. [40]	2015	Web-based	Web-based and a lecture-based cultural competency training	Likert-style multiple choice questions and short answer question	Cultural competencies are comparable

In three studies [32,34,40], students were assessed using the Likert scale and observed that e-content is an effective learning tool. In three studies [28,35,39], the evaluation was performed on the basis of MCQs and observed better performance and a better learning experience in the students. In another study [26], a standardized 44-point scale was used, which significantly improved student clinical performance. Two studies [27,37] used structured checklists and observed e-content as a preferred method. In one study [24], video links showed promising results, and students were evaluated based on their final examination scores. In four studies [23,29,36,38], a post-test was conducted as an assessment tool and showed promising results. Two studies [25,30] conducted quizzes and observed improved students' knowledge. One study [31] applied subjective, self-reported, and objective data as a method of assessment and observed significant improvement. A validated competency assessment rubric was used in a study [33] to assess surgical competence, and observed that due to the e-content intervention, the acquisition of surgical competency is rapid.

How Far Have We Come?

The amount of research being conducted and the technologies being developed are increasing rapidly; moreover, the rise in popularity of e-learning has also fueled this growth [41]. With this in mind, the field of e-content development will continue to grow at a rapid pace. With the rise in the adoption of digital platforms for education and healthcare, the demand for engaging and accessible content is expected to increase [41-43]. This means we expect e-content development to remain essential to medical teaching. Furthermore, with the growth of virtual, augmented, and mixed-reality platforms, the way we experience and consume digital content is also evolving. Since these new technologies are still evolving and gaining popularity, it is difficult to say how they will shape the future of e-content development [14,44]. However, it is safe to assume that e-content will be a significant part of the content ecosystem.

The Indian government has launched various initiatives for e-content development in medical education, such as the National Medical College Network (NMCN) project, which aims to connect all medical colleges in India through a common digital infrastructure and facilitate the creation and sharing of e-content [45,46]. The government has also established the National Digital Library of India (NDLI), which provides access to a wide range of e-resources, including e-books, e-journals, and other educational materials related to medical science [47].

Another important initiative is the National Program on Technology Enhanced Learning (NPTEL), which is a joint project by the Indian Institutes of Technology (IIT) and the Indian Institute of Science (IIS). NPTEL provides online courses and e-learning materials for various disciplines, including medical science, and has collaborated with several medical colleges and institutions to create e-content for their programs [48].

In addition to the aforementioned initiatives, the Indian government has also launched the Swayam platform, which offers free online courses from various Indian universities and institutions, including medical education. Swayam courses are designed to provide high-quality education to students who may not have access to traditional classroom learning and offer a flexible learning experience that students can complete at their own pace [49]. The government has also established the e-Granthalaya program, a digital library management software enabling medical colleges to create and manage their digital libraries. The program provides access to a wide range of e-resources, including e-books, e-journals, and other digital materials related to medical science [50].

The Indian government has also launched the National Health Stack, a digital infrastructure that aims to improve the quality and accessibility of healthcare services in India. The National Health Stack includes various components, such as a Health Identity Document (ID) system, a National Digital Health Mission, and a National Health Analytics Platform, which are designed to facilitate the creation and sharing of health data and e-content related to medical education with an objective of to establish a system of electronic health records based on global standards, freely available to the public, healthcare professionals, and service providers, and based on the consent of the public. The National Health Stack's Health ID system provides a unique digital identity to every citizen in India, which can be used to access their health records and other medical information. This system will also help create a National Electronic Health Registry that can be used for research, policy-making, and improving healthcare services in India [51].

Furthermore, the National Digital Health Mission aims to create a digital infrastructure for healthcare services in India, including creating a digital health ecosystem and a health data exchange that can be used in developing e-content for medical education [52]. The National Health Analytics Platform will also provide access to health data for researchers and policymakers, enabling them to make informed decisions and improve healthcare services in India [53]. Overall, these initiatives are helping to create a robust digital foundation for medical education and healthcare services in India, which will play a significant role in improving the overall health and well-being of the population.

Types of e-content

Video

Video is the most common type of e-content that are typically self-paced and can be viewed through a computer, phone, or tablet. Through a video, students can learn about diverse topics such as medical ethics and diseases [54]. Video-based learning provides an avenue to tackle a lot of educational issues. With more and more people owning mobile phones and online education platforms available to share information, there are incredible possibilities to use video content for medical schooling [55].

Audio

Audio is typically used for learning facts and figures or for recapping events that were covered in a lecture [56]. Through an audio, students can listen to content and learn from a teacher. To make e-content more impressive, audio should be included in the recordings. There are several free software options available, such as Free Sound Recorder, Audacity, and WavePad. Some of them are open source and allows to record live audio with several features. Recordings can be edited by cutting, copying, and mixing with sound effects. Moreover, the speed or pitch of the recording can be altered, or even old recordings can be converted into digital formats [57].

Figures

The notion of multimedia learning in cognitive theory suggests that comprehension is much deeper when utilizing words and visuals together rather than just words on their own. Historically, verbal instruction has been the primary means of teaching, including verbal and written forms. Nowadays, there are many visual learning materials available, yet merely adding pictures to words may not necessarily lead to better learning [58].

Simulation

This is an interactive type of e-content that can be used for learning or for simulated training. Taking the initiative to incorporate simulation-based medical education is a vital part of curriculum development [59]. Simulation is an encompassing term for a simulated recreation of a real-world process created with the purpose of facilitating learning through hands-on experience. Simulation-based medical education is defined as any educational activity that utilizes simulation aids to replicate clinical scenarios. Simulations are playing a huge part in undergraduate, and postgraduate training, medical professional development, emergency planning, and military trauma response [60]. In addition to utilizing simulation for teaching and training, it can also be used for summative assessment [61].

Quiz

These quizzes are typically used for assessing student knowledge or for review. Through a quiz, students can answer questions and receive feedback on their performance. Using quizzes to augment medical education is one such approach. The quiz has historically been utilized as a feedback assessment tool, but more recently, it has made its way into the medical curriculum, mainly informally. Medical quizzes often follow one of two formats: case-based or image-based. This method aids in bridging the knowledge gap between standard classroom instruction and clinical application [62]. The quiz is a simple tool that enhances didactic lectures by helping students learn and understand more. Being an interactive tool centered on students, it promotes regular feedback mechanisms and encourages active student participation. Web-based quiz games can also be used to summarize the key content [63].

Virtual Reality

This type of e-content is becoming increasingly popular as technology advances. Through virtual reality, students can explore a new environment or experience a situation that would be difficult to do in person [64]. Virtual reality is emerging as a new technique for presenting simulation. Benefits of virtual reality for educators and students include on-demand, affordable, repeatable, and standardized clinical instruction [65]. It has been observed that simulation is better than traditional clinical education in several areas and produces potent educational interventions that have both immediate and long-lasting effects [66].

These are just some of the types of e-content that are available today. As technology continues to evolve, more types of e-content will become available for use in education and training [67]. As e-content development becomes more popular, advancements are also being made in this field. This means that educators can expect to see even more benefits and advantages of e-content development in the future [68].

Advantages of e-content development in medical teaching

E-content development has a lot of potential and can be used in a variety of learning scenarios. While it initially gained popularity in higher education, it has since been applied to many other sectors, including healthcare [17,69,70]. In medical teaching, the advantages of e-content development are numerous. For example, it allows educators to deliver content in a more efficient and cost-effective manner [8,17]. Additionally, e-content can be updated quickly and easily, allowing educators to keep up with the latest developments in the field [71]. Furthermore, online materials can be accessed by students from anywhere in the world, enabling them to learn at their own pace [72]. Finally, e-content development allows educators to use a variety of interactive elements such as videos, quizzes, and simulations that can help to engage students and increase their learning retention rates [17,41]. Schools, hospitals, and healthcare providers have found that e-content development has helped to improve the learning experience for students [1,10]. With the rise in the adoption of digital platforms for education and healthcare, the demand for engaging and accessible content is expected to increase [73].

Digital content allows educators to create highly engaging learning experiences by incorporating multimedia, simulations, and interactive elements into their e-content [74,75]. For example, videos can streamline complex topics and diseases by highlighting important features or topics of an event. Moreover, the ability to deliver content to students in multiple formats and platforms has also expanded the reach and accessibility of e-content [76]. This means that students can now access the content they need on their preferred devices, such as tablets and smartphones, at any time. Furthermore, e-content also allows educators to create content that is easily accessible to all students regardless of their learning styles [77]. This is possible thanks to the use of basic language and design elements. Additionally, digital content can be easily updated and revised to include the latest and the most up-to-date information and resources [78]. This helps ensure that students are receiving the most up-to-date information.

Overall, digital content provides numerous benefits for educators and students alike. It allows educators to create highly engaging learning experiences that are accessible by all students [79]. Finally, it provides a platform for educators to reach a wider audience with their e-content. In this way, digital content is an invaluable resource for both educators and students alike [80]. Some of the advantages of e-content are described below (Table 2).

**Table 2:** Advantages of e-content development in medical education

Advantage	Description
Flexibility and Convenience	E-content can be accessed anytime and anywhere, making it easier for medical students to learn at their own pace and on their own schedule [17].
Interactive Learning	E-content allows for the integration of multimedia elements, such as videos, images, and simulations, that can enhance the learning experience and promote greater engagement [12].
Cost-Effective	E-content development can be more cost-effective than traditional classroom-based instruction, as it eliminates the need for physical materials and resources [71].
Personalized Learning	E-content can be tailored to meet the specific needs of individual students, allowing for personalized learning experiences that can improve learning outcomes [81].
Improved Retention and Recall	The interactive and engaging nature of e-content can help students better retain and recall information, leading to improved learning outcomes [82].
Continuous Improvement	E-content can be easily updated and revised, allowing medical educators to quickly adapt to new information and changes in the field [83].
Collaboration and Communication	E-content development can facilitate collaboration and communication among medical students and educators, allowing for more efficient and effective knowledge-sharing [32].
Accessibility and Inclusivity	E-content can be made accessible to a wider audience, including students with disabilities, and can be translated into multiple languages, making medical education more inclusive [84,85].
Efficient Assessment	E-content can include built-in assessments and quizzes, allowing medical educators to quickly and efficiently evaluate student learning and identify areas for improvement [86].
Sustainability	E-content is a more environmentally sustainable option than traditional classroom-based instruction, as it eliminates the need for physical materials and resources [87].

Challenges of e-content development in medical teaching

Medical education utilizes traditional teaching methods such as lectures, which can become ingrained in an organization's culture. This can lead to a reluctance to adopt new technologies [74]. As e-content development becomes more popular, it is also experiencing some challenges. For example, while virtual environments offer many benefits, they can also be expensive and difficult to implement [76]. Moreover, while some types of simulations can be quite effective, they can also be quite challenging to implement [88]. This means that while e-content development can be quite effective, it is important to understand the various challenges that it faces. Another challenge that many educators face when developing e-content is that it can be difficult for them to stay on track. This can be especially challenging for educators who are also responsible for teaching students face-to-face [89]. With the increasing use of high-tech devices for learning, it is important for educators to stay up-to-date with technology [41].

E-content is limited in that it cannot be used to assess student reactions face-to-face. However, video conferencing tools can help overcome this limitation [11]. Lastly, creating content that is engaging and relevant to a wide range of learners can be difficult [90]. This means that educators need to be creative in order to make sure their content is effective and useful for students. Limited access to technology, despite the increasing availability of technology, many parts are still lack access to reliable internet connectivity, computers, and other devices required for e-learning [91]. Some major disadvantages or limitations are described below (Table 3).

**Table 3:** Limitations in e-content development

Limitations	Description
Language barriers	In a diverse county like India with many different languages spoken, which can make it challenging to develop e-content that is accessible to all students [92].
Quality control	The quality of e-content for medical education can be inconsistent, with some materials being of low quality or inaccurate [93,94].
Cost	Developing high-quality e-content can be expensive, and many educational institutions in developing or in underdeveloped countries may not have the financial resources to invest in it [95,96].
Lack of standardization	There is a lack of standardization in e-content development for medical education, which can lead to confusion among students and faculty [97].
Limited interaction	E-content may not provide the same level of interaction as traditional classroom-based education, which can limit students' ability to ask questions and engage in discussions [75].
Inadequate teacher training	Teachers may not be adequately trained to develop and use e-content, which can limit the effectiveness of online learning [89,98].
Lack of motivation	Some students may lack the motivation to engage with e-content, which can reduce the effectiveness of the learning experience [99,100].
Plagiarism and copyright issues	E-content development can be hindered by issues related to plagiarism and copyright infringement, which can lead to legal problems and damage the credibility of the educational institution [101].

Future of e-content development in medical teaching

It is obvious that a lot has been achieved in the domain of electronic content creation, however, there is still a lot more to do. This means that while the field has seen significant growth and progress in the past decade, it is also expected to continue evolving in the future [17]. With this in mind, it can be expected that e-content development to continue playing an important role in medical teaching [93]. In addition to the benefits that were discussed in this article, the use of e-content has also allowed educators to make use of more diverse technologies. As technology evolves and becomes more accessible, e-content will be used even more [67]. With this in mind, it is expected that the use of e-content in medical teaching will continue to increase in the future.

The future of e-content development in medical teaching is likely to see continued growth and innovation as technology advances and more educators and learners recognize the benefits of online and digital resources [102,103]. This may include the development of interactive and immersive educational experiences, such as virtual reality simulations and gamification, as well as the integration of artificial intelligence and machine learning to personalize learning and provide real-time feedback [104]. However, it will also be important to ensure that these e-content developments are accessible, inclusive, and evidence-based and that they complement rather than replace traditional teaching methods [105].

## Conclusions

Electronic content production is a relatively new discipline; however, its expansion is occurring extremely quickly. The e-learning sector is expanding, and e-content development has a lot of potential and can be used in various learning scenarios. It can be used for flipped classrooms, blended learning, and virtual classrooms. It can also be used to supplement existing instructional materials or to create new ones. E-content development has numerous benefits for medical educators and their students. The most important of these is the ability to deliver content to students in an engaging and easy-to-understand manner. Digital content allows educators to create highly engaging learning experiences by incorporating multimedia, simulations, and interactive elements into their e-content.
